# Near-medians that avoid the corners; a combinatorial probability approach

**DOI:** 10.1186/1471-2164-15-S6-S1

**Published:** 2014-10-17

**Authors:** Caroline Anne Larlee, Chunfang Zheng, David Sankoff

**Affiliations:** 1 Department of Mathematics and Statistics, University of Ottawa, 585 King Edward Avenue, Ottawa, Canada K1N 6N5

**Keywords:** median problem, breakpoint distance, combinatorial probabilities

## Abstract

**Background:**

The breakpoint median for a set of *k ≥ *3 random genomes tends to approach (any) one of these genomes ("corners") as genome length increases, although there are diminishing proportion of medians equidistant from all *k *("medians in the middle"). Algorithms are likely to miss the latter, and this has consequences for the general case where input genomes share some or many gene adjacencies, where the tendency for the median to be closer to one input genome may be an artifact of the corner tendency.

**Results:**

We present a simple sampling procedure for constructing a "near median" that represents a compromise among *k* random genomes and that has only a slightly greater breakpoint distance to all of them than the median does. We generalize to the realistic case where genomes share varying proportions of gene adjacencies. We present a supplementary sampling scheme that brings the constructed genome even closer to median status.

**Conclusions:**

Our approach is of particular use in the phylogenetic context where medians are repeatedly calculated at ancestral nodes, and where the corner effect prevents different parts of the phylogeny from communicating with each other.

## Background

**Figure 1:**
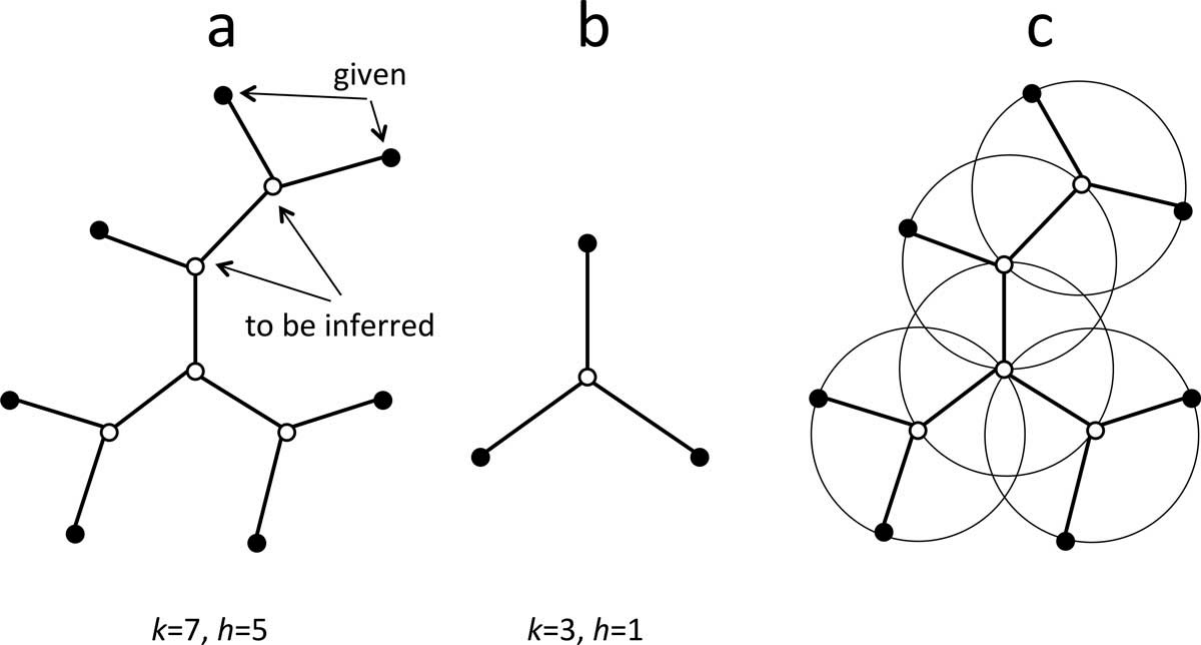
**Small phylogeny and median problems**. a. Small phyogeny problem. Open dots represent ancestral nodes with unknown position in the metric space. Black dots are at given positions in the metric space. b. Median problem, with three given points and one to be inferred. c. Decomposition of the small phylogeny problem into several overlapping median problems to be solved simultaneously. N.B. open nodes may have degree *≥ *3.

The *small phylogeny problem *is a familiar model for evolutionary biology: given a graph theoretical tree *T *with *k ≥ *3 vertices of degree 1 (*terminal, observed *or *present-day *nodes), each associated with a point in some metric space, and *h ≥ *1 vertices of degree 3 or higher (*non-terminal, hypothetical *or *ancestral *nodes), it is required that each of *h *ancestral nodes be associated with some point in the metric space so as to minimize the sum of the distances over all pairs of adjacent vertices in *T*. This is illustrated in Figure 1a. The prototypical small phylogeny problem is the case of *k *= 3 and *h *= 1. This is called the *median problem *and is illustrated in Figure 1b. The minimum sum of the distances between the ancestor and its adjacent vertices is called the *median score*.

The "steinerization" approach to the small phylogeny problem, dating from 1976 in sequence space [1] and from 1997 in gene order space [2], is illustrated in Figure 1c. We decompose *T *into *h *overlapping median problems, each problem focusing on one of the *h *ancestral points and the three or more vertices of *T *that are adjacent to it. Suppose we have a way of solving the median problem. After initializing the ancestral points in *T *randomly or in some other way, the ancestral positions can be improved one at a time. For each ancestral node, we simply apply the median solution to the current positions of its immediate neighbors. At each step the new median is retained only if it has a lower score than the current value. The process is iterated until no further improvement is possible. The output, not necessarily unique and not necessarily optimal, where every ancestor vertex is at the median position of its adjacent vertices, is called a "steinerized" solution for the small phylogeny problem, after the well-known Steiner problem in Euclidean 2-space.

The value of the median as a prototype and as a component step for the construction of gene-order phylogenies has been undermined by simulations that show the median for a set of *k ≥ *3 random input genomes tends, as genome length *n *increases, to coincide with any one of these *k *genomes themselves [3,5]. The median thus reflects no gene-order information from any of the other *k − *1 genomes: the "medians in the corner" effect. There are some medians that are "in the middle", containing information drawn from several, or all, the *k *genomes, but these become relatively rare as genome length increases [3]. Because these observations come from simulations followed by application of complex optimization algorithm [4], we can derive no precise analytic solutions about the probabilities of different kinds of median. These observations hold for all kinds of genomes, signed, unsigned, with single or multiple (with a bounded number of chromosomes), circular or linear chromosomes, and for all kinds of genomic distance: e.g., breakpoint distance and double-cut-and-join distance.

How important these findings are, for sets of genomes that have substantial gene order commonalities, is not clear. Even for random genomes, not only do we observe some medians in the middle, but any fixed genome can be shown to be the median, or close to the median, of *k ≥ *3 other genomes that are essentially random with respect to each other.

The corner tendency has serious implications for "small phylogeny" analyses using a steinerization strategy, where each ancestral node in a given unrooted tree is the median of its three neighbours. Here, an ancestral genome is determined by iterating a median algorithm over the tree, starting with arbitrary initial genomes. When medians tend to fall at or near corners, the iteration process cannot effectively transfer relevant genomic commonalities between remote branches of the tree. How can we avoid this pathology in a principled way?

In this paper, we propose a simple initial construction for a genome which includes gene-order information from all the *k *given genomes. These are "near medians" in a well-defined sense, and they approximate true medians as *k *increases, and as the common gene-order information between subsets of the genomes increase. Because the construction is based on a binomial sampling scheme, we can analytically derive quantitative predictions about all the results. A second sampling step, somewhat more complicated, then substantially improves the initial construction.

## Results

### The basic construction

Consider three signed genomes, I, II and III, each consisting of one or more circular chromosomes, containing the same *n *genes and each containing *n *gene adjacencies. Since the genomes are signed, the genes have polarity, from the *− *end to the + end, or from + to *−*. Each adjacency is thus an unordered pair of the 2*n *gene ends, chosen from among $(\begin{array}{c} 2n\\ 2 \end{array})$ possibilities. The analysis is essentially the same for linear, circular, unichromosomal or multichromosomal genomes; the effect of allowing a bounded number *>*1 of chromosomes would be *o*(*n*) as would be the differences between circular and linear models. We assume that the genomes are randomly ordered, which means, for all intents and purposes, that they share virtually no adjacencies; the expected number of shared adjacencies is actually 0.5, a constant, even for very large *n*.

**Figure 2:**
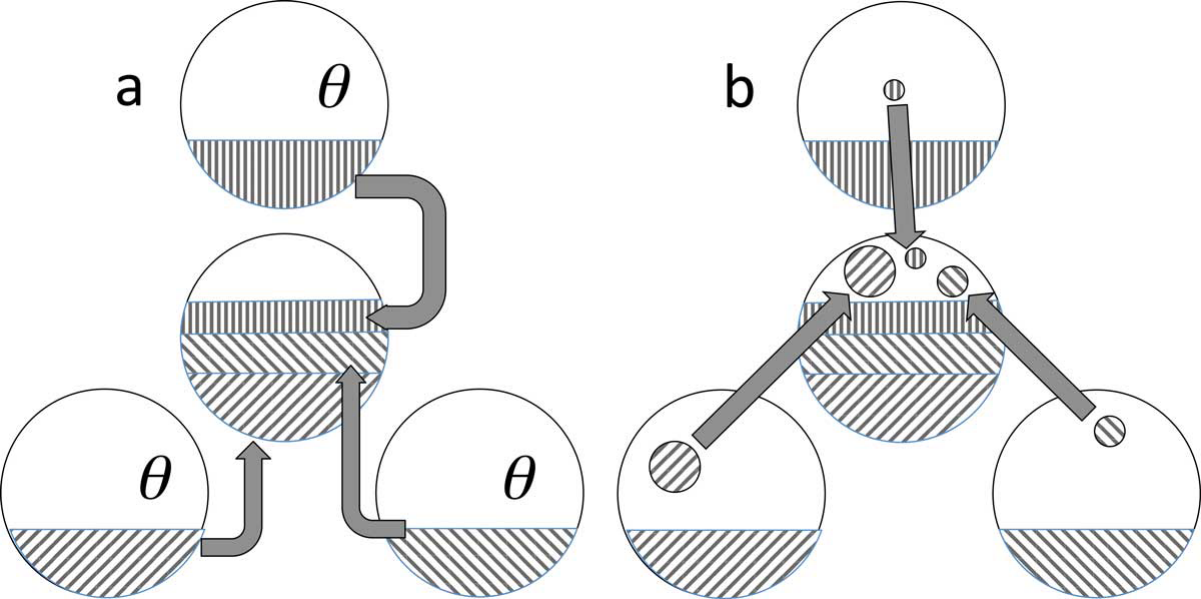
**Two stage sampling scheme**. a. First sampling of *θn *adjacencies from each of three genomes. b. Supplementary sampling of residual adjacencies consisting of two free ends.

Motivated by the search for a median in the middle, we construct a set  A containing *n *adjacencies containing comparable amounts of information from each of genomes I, II and III. As a first step, we wish to select the same number of adjacencies *θn *from each of the three genomes, as in Figure 2a. Obviously, $\theta \leq \frac{1}{3}$. Randomly select *θn *adjacencies from genome I. Turning our attention to genome II, the expected proportion of "two free ends", adjacencies where neither end appears in a previously selected genome I adjacency, is

(1) $${\left(1-\theta \right)}^{2}\geq \frac{4}{9}>\frac{1}{3}\geq \theta .$$

Thus we can expect ample adjacencies in genome II from which to pick *θn *which do not conflict with any of those selected from genome I.

Similarly, having then selected *θn *pairs from each of genomes I and II, the expected proportion of pairs in genome III with two free ends is (1 *− *2*θ*)^2^.

We need at least *θn *such pairs to contribute the same number as the other two genomes. At the level of expectations,

(2) $${\left(1-2\theta \right)}^{2}\geq \theta .$$

Thus the maximum value of *θ *should be less than the root of

(3) $$1-5\theta +4\theta^{2}=0,$$

so $\theta \leq \frac{1}{4}$. Setting $\theta \leq \frac{1}{4}$ produces a set $A^{\prime }$ of at most $\frac{3}{4}n$ compatible adjacencies, drawn equally from genomes I, II and III. Compatibility simply means that no two adjacencies contain the same gene end. The value $\frac{3}{4}n$ is attainable by fixing $\theta =\frac{1}{4}$.

We can construct an additional $\frac{n}{4}$ compatible adjacencies to bring $A^{\prime }$ up to full genome status (containing linear and/or circular chromosomes), simply by using any (graph-theoretical) matching on the $\frac{n}{2}$ gene ends that do not occur in $A^{\prime }$.

The breakpoint distance between two genomes can be defined as *D *= *n − a*, where *a *is the number of adjacencies they contain in common. For a genome with set of adjacencies  A, the sum of the normalized distances to the three input genomes,

(4) $$\frac{1}{n}\underset{\text{genome}\;\text{G}\;=\;\text{I},\;\text{II},\;\text{II}}{\sum }D\left(A,\;\text{genome}\;G\right),$$

is called its score. With $\theta =\frac{1}{4}$, the score of  A is *≤ *2.25.

The second step in the construction of  A may be done in such a way as to decrease its score below 2.25, but this depends on the input genomes as well as our initial sampling of *θ *adjacencies per genome, so we cannot make statistical predictions as easily as we can for the first step. The second step will be discussed in detail later in this paper.

It is remarkable that by blindly sampling adjacencies, with no view towards optimization, we can construct a genome which has a score of 2.25 or less, when a median would have a score of 2.0, not that much smaller. Moreover, $A^{\prime }$ represents each input genome equally, so  A is close to the "middle", whereas a median found through optimization is more likely to be in a "corner", especially as *n *increases. In addition, as we detail in the next subsection, we can obtain the probability distribution of properties of $A^{\prime }$ analytically. Finally and most important, this basic construction is the key to a number of other developments that we will detail later in this paper.

### Statistical properties of the construction

Once the selection of *θn *adjacencies has been made in genome I and genome II, the probability that *X *of *n *adjacencies in genome III each contains two free ends has a binomial probability *B*(*X*; *n, p_θ_*) where *p_θ _*= (1 *− *2*θ*)^2^. Thus

(5) $$B\left(X;n,p_{\theta }\right)=\left(\begin{array}{c} n\\ X \end{array}\right){\left(1-2\theta \right)}^{2X}{\left(4\theta \left(1-\theta \right)\right)}^{n-X}.$$

The standardized normal approximation to *X *is

(6) $$Z=\frac{X+0.5-n{\left(1-2\theta \right)}^{2}}{\sqrt{n{\left(1-2\theta \right)}^{2}4\theta \left(1-\theta \right)}},$$

taking into account the continuity correction (*±*0.5).

Table 1 shows the probability that the construction of sample of *θn *adjacencies can be carried out, based on the normal distribution constructed in Equation (6). Of note is the phase change at *θ *= 0.25, corresponding to our solution of Equation (3).

**Table 1:** Probability of at least *θn *adjacencies with two free ends in genome III.

	*θ *: 0.10	0.20	0.24	0.25	0.26	0.30	0.333
*n *= 5	0.9798	0.6101	0.3630	0.3028	0.2457	0.0716	0.0109
10	0.9994	0.7657	0.4445	0.3575	0.2750	0.0506	0.0031
50	1	0.9864	0.6273	0.4351	0.2530	0.0026	0
100	1	0.9994	0.7163	0.4540	0.2056	0	0
500	1	1	0.9761	0.4794	0.0521	0	0
1000	1	1	0.9834	0.4854	0.0119	0	0
2000	1	1	0.9988	0.4897	0.0008	0	0
10000	1	1	1	0.4954	0	0	0

**Figure 3:**
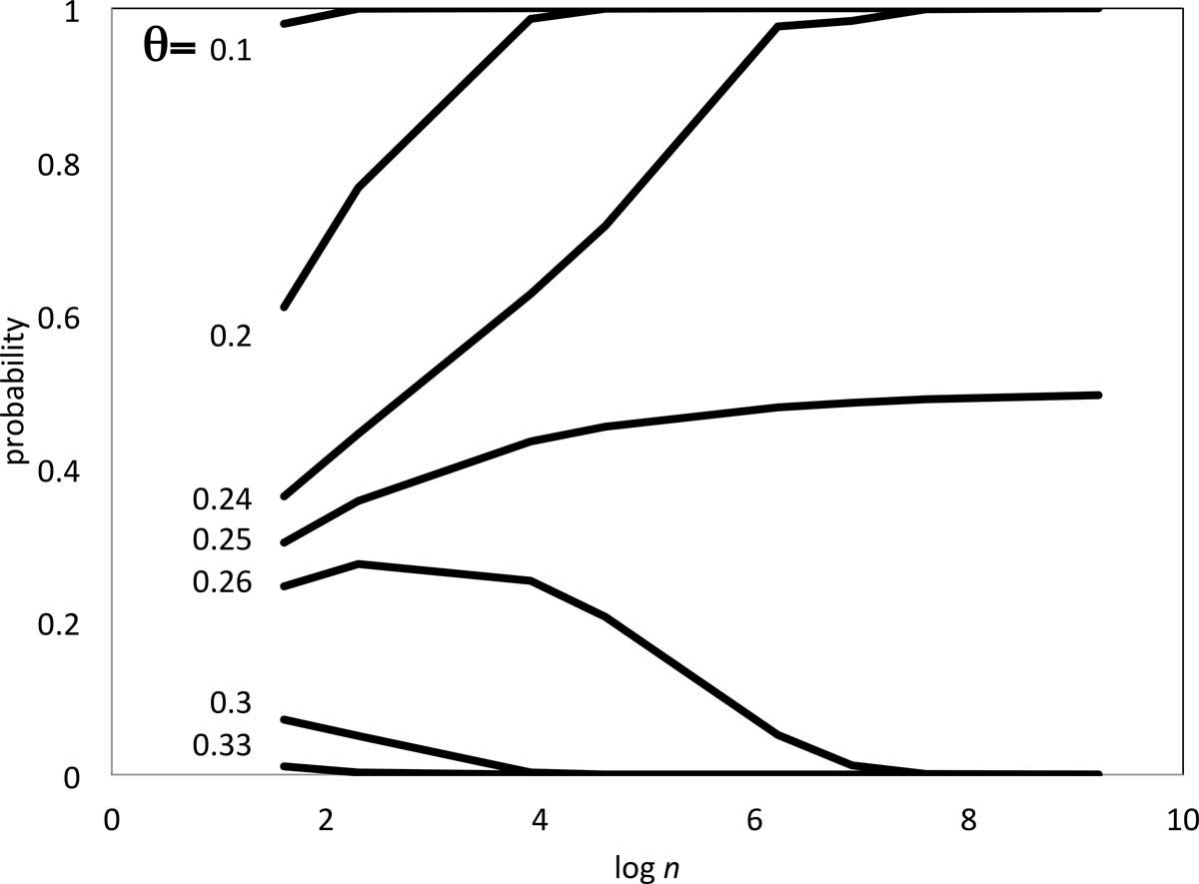
**Phase change at *θ *= 0.25**. Probability of at least *θn *adjacencies with two free ends in genome III.

These results are also illustrated in Figure 3.

The case of *k >*3 genomes

When there are *k *= 4 input genomes, inequality (2) is replaced by

(7) $${\left(1-3\theta \right)}^{2}\geq \theta$$

and Equation (3) by

(8) $$1-7\theta +9\theta^{2}=0.$$

The root of this is *θ *= 0.18858, so that $\frac{\left|A^{\prime }\right|}{n}=0.75432$, leading to an  A having a score, the sum of the normalized distances to the four input genomes, of at most 3.24568, compared to the median score of 3.0 [3].

For a general *k ≥ *3 the maximum value of *θ *must satisfy

(9) $${\left(1-\left(k-1\right)\theta \right)}^{2}\geq \theta$$

and must then be the root of

(10) $$1+\theta \left(1-2k\right)+\theta^{2}\left(1-2k-k^{2}\right)=0.$$

Thus

(11) $$\theta =\frac{2k-1-\sqrt{4k-3}}{2\left(1-2k+k^{2}\right)}.$$

Note that that for *k ≥ *3 genomes drawn at random, the expected normalized median sum will be equal to *k − *1 [3]. This score of a genome constructed with our method is

(12) $$k(1-\frac{2k-1-\sqrt{4k-3}}{2\left(1-2k+k^{2}\right)})\to k-1,$$

as *k → ∞*. An impression of the speed of convergence is given in Table 2, calculated using the normal approximation.

**Table 2:** Approach of normalized sum of distances to median score, with increasing *k*.

*k - *1	∑ *D*
2	2.25
3	3.2457
4	4.2375
9	9.2026
49	49.1153
99	99.0864
499	499.0419
999	999.0302
1999	1999.0216
9999	9,999.0099

### Nonrandom genomes

We have hitherto analyzed the case of genomes that are completely random with respect to each other. In practice this is of limited interest, but the techniques and bounds developed for this case are essential to more realistic situations where an input genome shares a non-trivial proportion of its adjacencies with some or all of the other input genomes.

Suppose all three genomes share *ψn *adjacencies. If we have selected, from each of two genomes containing the same *n *genes, *θn *+ *ψn *adjacencies, the number of remaining adjacencies with two free ends in the third genome is (1 *− *2*θ − ψ*)^2^*n*.

We need at least *θn *such adjacencies to contribute equally as the other two genomes. Thus

(13) $${\left(1-2\theta -\psi \right)}^{2}\geq \theta .$$

The maximum value of *θ *is the root of

(14) $$4\theta^{2}+\theta \left(4\psi -5\right)+\psi^{2}-2\psi +1=0,$$

namely

(15) $$\theta =\frac{-4\psi +5-\sqrt{9-8\psi }}{8},$$

in which case the score of  A is at most

(16) $$3\left(1-\left(\frac{-4\psi +5-\sqrt{9-8\psi }}{8}+\psi \right)\right).$$

Similarly, for any *k ≥ *3 input genomes all sharing *ψn *adjacencies, *θ *satisfies

(17) $$\theta \leq \frac{2k+2\psi -2k\psi -1-\sqrt{-3+4\left(k+\psi -k\psi \right)}}{2{\left(k-1\right)}^{2}}$$

and the score of  A is at most

(18) $$k\left(1-\left(\frac{2k+2\psi -2k\psi -1-\sqrt{-3+4\left(k+\psi -k\psi \right)}}{2{\left(k-1\right)}^{2}}+\psi \right)\right).$$

The median score for this case is

(19) $$k\left(1-\left(\psi +\frac{1-\psi }{k}\right)\right).$$

Comparing the expressions (18) and (19), as *k *gets larger, the two scores become closer and closer. Table 3 illustrates how our construction converges to the median as *k *increases, for one value of *ψ *= 0.625.

**Table 3:** Approach to true median for *ψ *= 0.625 as *k *increases

*k*	distance	true median value	difference
3	0.9375	0.750	0.1875
4	1.2989	1.125	0.1739
5	1.6634	1.500	0.1634
10	3.5067	3.375	0.1317
50	18.4468	18.375	0.0718
100	37.1785	37.125	0.0535
500	187.1507	187.125	0.0257
1000	374.6435	374.625	0.0185
2000	749.6383	749.625	0.0133
10,000	3,749.6310	3,749.625	0.0060

In the most general case for three input genomes, suppose all three genomes share *ψn *adjacencies and each pair of genomes (*i, j*)*, i *≠ *j *share an additional number *ω_i,j_*, where for *i *≠ *h *≠ *j*, we require

(20) $$\psi +\omega_{i,j}+\omega_{i,h}\leq 1.$$

Note that in this case, there may be some asymmetry among the three genomes.

At the outset, all the adjacencies shared among at least two genomes are included in constructing  A. We then select from each of two genomes *θn *adjacencies from those that remain. The number of remaining adjacencies with two free ends in the third genome is ${\left(1-2\theta -\psi -\sum_{i\neq j}\omega_{i,j}\right)}^{2}n$. We need at least *θn *such adjacencies to contribute equally, so

(21) $${\left(1-2\theta -\psi -\underset{i\neq j}{\sum }\omega_{i,j}\right)}^{2}\geq \theta ,$$

where *θ *is constrained by $\theta +\psi +\sum \omega_{i,j}\leq 1$, which is stronger than condition (20). The maximum value of *θ *must be the root of

(22) $$4\theta^{2}+\theta \left(-5+4\;\underset{i\neq j}{\sum }\omega_{i,j}+4\psi \right)-2\left(\underset{i\neq j}{\sum }\omega_{i,j}+\psi \right)+{\left(\underset{i\neq j}{\sum }\omega_{i,j}+\psi \right)}^{2}=0.$$

Hence

(23) $$\theta =\frac{5-4\left(\sum_{i\neq j}\omega_{i,j}+\psi \right)-\sqrt{9-8\left(\sum_{i\neq j}\omega_{ij}+\psi \right)}}{8}.$$

and the score is

(24) $$3\left(1-\left(\frac{5-4\left(\sum_{i\neq j}\omega_{i,j}+\psi \right)-\sqrt{9-8\left(\sum_{i\neq j}\omega_{ij}+\psi \right)}}{8}+\psi +\underset{i\neq j}{\sum }\omega_{i,j}\right)\right).$$

### Improving the set of compatible adjacencies

Consider our original construction where genomes I, II and III, with no adjacencies in common, each contribute $\frac{n}{4}$ adjacencies to make up $A^{\prime }$. Clearly there must be $\frac{n}{2}$ gene ends, out of a total of 2*n*, not selected for inclusion in $A^{\prime }$. We earlier suggested that any matching of these genes ends could be added to $A^{\prime }$ to make up a full genome. In fact this can be done in such a way as to decrease the score of  A below 2.25.

We revisit the selection of $\theta n=\frac{1}{4}n$ adjacencies from the three genomes. After selecting these from genome I, a proportion $1-\theta =\frac{3}{4}$ of the gene ends will remain free, namely $\frac{3}{2}n$ ends. This means that in each of genomes II and III, $\theta^{2}=\frac{1}{16}$ of the adjacencies will have had two ends already selected, $2\theta \left(1-\theta \right)=\frac{6}{16}$ will have one free and one selected and ${\left(1-\theta \right)}^{2}=\frac{9}{16}$ will have two free ends. After selecting $\theta n=\frac{1}{4}n$ adjacencies from the $\frac{9}{16}n$ to contribute to $A^{\prime }$, we are left with $\frac{5}{16}n$ with two free ends in genome II. These ends are individually available during the selection of adjacencies from genome III or, eventually, for the supplementary sampling.

At the same time, of the $2\left(1-\theta \right)=\frac{3}{2}n$ free ends left after the genome I selection, only $2\left(1-\theta \right)n-2\theta n=\frac{3}{2}n-2\times \frac{1}{4}n=n$ ends still remain free after the genome II selection. In other words $\frac{2}{3}$ of the $\frac{3}{2}n$ originally free genome ends still remain free. Of the $\frac{3}{4}n$ adjacencies originally with two free ends in genome I, only $\frac{3}{4}{\left(\frac{2}{3}\right)}^{2}n=\frac{1}{3}n$ still have two free ends. These are available for selection from genome III or, eventually, for the supplementary sampling.

Now consider the $\frac{9}{16}n$ adjacencies left in genome III after selecting the $\theta =\frac{1}{4}n$ from genome I. Of these only $\frac{9}{16}{\left(\frac{2}{3}\right)}^{2}n=\frac{1}{4}n$ will have two free ends, and all of these must be contributed to $A^{\prime }$.

These adjacencies contain $\frac{n}{2}$ free ends. But we know that genome III contained *n *free ends before contributing $\frac{n}{2}$ to $A^{\prime }$. Thus after the contribution of $3\theta n=\frac{3}{4}n$ adjacencies to $A^{\prime }$ by the three genomes, there remain $\frac{n}{2}$ free ends in genome III, all of which are adjacent to selected ends, and cannot participate in supplementary contributions to construct  A. Because the $\frac{n}{2}$ ends are randomly distributed among the adjacencies in genomes I and II, as are the $\frac{n}{2}$ selected for contribution to $A^{\prime }$, in the supplementary sampling we know there remain $\frac{1}{3}n$ free ends available in genome I and $\frac{5}{16}n$ in genome II. Thus

(25) $$\frac{1}{3}{\left(\frac{1}{2}\right)}^{2}n+\frac{5}{16}{\left(\frac{1}{2}\right)}^{2}n=\frac{31}{192}n$$

adjacencies are available to be added to $A^{\prime }$ to construct  A with a better score than 2.25.

Some of these adjacencies may conflict, i.e., share an end, so the actual reduction in the score must be found by maximum matching and will differ substantially from one instance to another. Simulations show that the average number of compatible adjacencies is about $\frac{3}{4}\times \frac{31}{192}n$. The score of  A, once additional adjacencies not in genomes I, II or III have been formed by matching the remaining free ends, thus completing the set of *n *adjacencies, has been reduced to around 2.13, which is closer to the median value of 2.0.

We have thus constructed something very close to the median in two stages, the first being the random sampling of $\theta n=\frac{1}{4}$ compatible adjacencies from each of the three genomes. The second stage is the search, in all of the genomes, for residual adjacencies where neither end has been chosen in the first stage, as in Figure 2b.

## Conclusions

In this paper, we started with a simple construction of a set of adjacencies drawing equally on three random genomes, in an effort to avoid degenerate medians consisting of one of the input medians. This construction produced a normalized sum of distances score of 2.25 instead of the median value of 2. (See reference [5] for a proof of the median value.) We then generalized this to *k ≥ *3 input genomes, leading to better and better approximations to the median value of *k − *1.

The results for *k *= 3 could then be generalized to the more realistic situation where the genomes have adjacencies in common.

Finally for the case of *k *= 3, we found a way of improving the construction to come within 6-7% of a median genome, containing roughly equal contributions from each of the inputs.

It should be noted that the basic construction we start out with is severely non-unique, since it is based on one random sample among ${\left(\begin{array}{c} n\\ \frac{n}{4} \end{array}\right)}^{3}$ possibilities. On the other hand, it does contain information about all three input genomes, rather than just one as in the case of a corner median. In realistic situation where the input genomes have some or many adjacencies in common, these are all captured (for *k *= 3, at least) and the basic construction only organizes the rest of the near-median.

The sampling we describe here runs in time linear with *n*, including the supplementary sampling as long as each gene end carries sufficient information. If a maximum matching algorithm is used at the end, this is theoretically *O*(*n*^3^), but in practice only a small number of gene ends are involved at this step. This contrasts with classical median solvers, e.g., [6], which are severely exponential in running time, especially for genomes that are highly rearranged with respect to each other.

We propose that near-medians may be more useful than corner medians in the phylogenetic context, lacking any technical capacity to detect medians in the middle (also non-unique) for large *n *without costly multiple runs of median solvers.

## Competing interests

The authors declare that they have no competing interests.

## Authors' contributions

All authors carried out the research, wrote the paper, read and approved the manuscript.

## Declarations

The publication charges for this article were funded by the Canada Research Chair in Mathematical Genomics, and by the University of Ottawa.

This article has been published as part of *BMC Genomics *Volume 15 Supplement 6, 2014: Proceedings of the Twelfth Annual Research in Computational Molecular Biology (RECOMB) Satellite Workshop on Comparative Genomics. The full contents of the supplement are available online at http://www.biomedcentral.com/bmcgenomics/supplements/15/S6.

## References

[B1] Sankoff D Cedergren RJ Lapalme J Frequency of insertion-deletion, transversion, and transition in the evolution of 5S ribosomal RNA Journal of Molecular Evolution 1976 7 133 149 10.1007/BF01732471 772222

[B2] Sankoff D Blanchette M Multiple genome rearrangement and breakpoint phylogeny Journal of Computational Biology 1998 5 555 570 10.1089/cmb.1998.5.555 9773350

[B3] Haghighi M Sankoff D Medians seek the corners, and other conjectures BMC Bioinformatics 2012 13 S19:S5 10.1186/1471-2105-13-195 PMC3526443 23281922

[B4] Boyd S Haghighi M A fast method for large-scale multichromosomal breakpoint median problems Journal of Bioinformatics and Computational Biology 2012 10 1240008 10.1142/S0219720012400082 22809309

[B5] Jamshidpey Arash Jamshidpey Aryo Sankoff D Sets of medians in the non-geodesic pseudometric space of unsigned genomes with breakpoints BMC Genomics 2014 15 Suppl 6 S3 10.1186/1471-2164-15-S6-S3 PMC4240729 25571965

[B6] Xu AW A fast and exact algorithm for the median of three problem: A graph decomposition approach Journal of Computational Biology 2009 16 1369 1381 19747038 10.1089/cmb.2009.0087

